# Homeopathy “Putting on Airs”

**Published:** 1868-05

**Authors:** 


HOMEOPATHY "PUTTING ON AIRS."—Dr. F. H. Krobs, of Boston, recently read a protest against a woman being admitted as member of the Massachusetts Homeopathic Medical Society, and quoted several texts of Scripture to show that woman was, by divine law, expected to occupy a subordinate place in human society—that she was not to teach or usurp authority, but to keep silence in the churches. He thought her deficient in talent for the abstract sciences, lacking originality and genius, and affirmed that women, when misplaced, became bold, arrogant, tyrannical and full of folly. The society voted thirty-one for, and thirty-three against, admission.

